# Significance and potential of marine microbial natural bioactive compounds against biofilms/biofouling: necessity for green chemistry

**DOI:** 10.7717/peerj.5049

**Published:** 2018-06-27

**Authors:** Mohd Adnan, Eyad Alshammari, Mitesh Patel, Syed Amir Ashraf, Saif Khan, Sibte Hadi

**Affiliations:** 1 Department of Clinical Laboratory Sciences, College of Applied Medical Sciences, University of Hail, Hail, Saudi Arabia; 2 Department of Clinical Nutrition, College of Applied Medical Sciences, University of Hail, Hail, Saudi Arabia; 3 Department of Biosciences, Bapalal Vaidhya Botanical Research Centre, Veer Narmad South Gujarat University, Surat, India; 4 School of Forensic and Applied Sciences, University of Central Lancashire, Preston, UK

**Keywords:** Biofouling, Biofilm, Bioactive compounds, Green chemistry, Marine microbes

## Abstract

Natural products from the unique environments of sea water and oceans represent a largely unfamiliar source for isolation of new microbes, which are potent producers of secondary bioactive metabolites. These unique life-forms from the marine ecosphere have served as an important source of drugs since ancient times and still offer a valuable resource for novel findings by providing remedial treatments. Therefore, it can be expected that many naturally bioactive marine microbial compounds with novel structures and bioactivities against those from terrestrial environments may be found among marine metabolites. Biofilms in aquatic environment possess serious problems to naval forces and oceanic industries around the globe. Current anti-biofilm or anti-biofouling technology is based on the use of toxic substances that can be harmful to their surrounding natural locales. Comprehensive research has been done to examine the bioactive potential of marine microbes. Results are remarkably varied and dynamic, but there is an urgent need for bioactive compounds with environmentally friendly or “green” chemical activities. Marine microbes have the potential as upcoming and promising source of non-toxic compounds with sustainable anti-biofouling/anti-biofilm properties as they can produce substances that can inhibit not only the chemical components required for biofilm production but also the attachment, microorganism growth, and/or cell–cell communication.

## Introduction

Little is known about the ecology of marine microorganisms, which is probably the reason that attention was not given by chemists and ecologists to these organisms for many years. As they flourish in diverse types of ecological pressures, climates, food supplies, and darkness, these marine organisms develop certain adaptation mechanisms. This can include the struggle for space, avoidance of predators, ability to effectively reproduce, and many other unknown defense mechanisms. Evolution/production of unique and natural bioactive metabolites is one such outcome resulting from these adaptations, which can be beneficial for human beings in many ways. They can possibly be responsible for the interaction with chemical components of biofilms (Joint, Muhling & Querellou, 2010).

Naturally bioactive chemical compounds produced by bacteria and diatoms can cause disruption in biofilm formation (Ganapiriya, Maharajan & Kumarasamy, 2012); therefore, they can be useful in development of environmentally friendly compounds for protection against marine bio-fouling (Holmström & Kjelleberg, 1999**)**. It is just a matter of finding the correct naturally bioactive compound for a specific application. Coating and application of active ingredients from marine organisms (invertebrates, microorganisms, algae) that prevent the growth and settlement of fouling organisms has been proposed since the 1980s (Maki et al., 1988) but none of the potent, non-toxic, naturally bioactive compounds from diverse marine life-forms are found to be active against biofouling. When compared with the natural anti-biofoulants from macro-organisms (sponges, mollusks, tunicates, bryozoans, polychetes, and many other marine invertebrates), very limited information is available from marine microorganisms (Fusetani, 2004).

Our aim through this literature review was to search for potential biologically active compounds, whose chemical information can be useful in facilitating the development of new anti-biofouling or anti-biofilm agents from natural marine sources (Li et al., 2018). These agents should possess some extent of bioactivity, either against biofilms or any other biofilm producing microorganism and must rely on two actions: (1) synergistic action and (2) continuous production of enough potent molecules/defensive compounds by microbes so that they may also be induced upon attacks on the host. Thus far, after comparing the available information on bioactive metabolites from macro-organisms, only a small number of microorganisms have been investigated for bioactive metabolites (Debbab et al., 2010). This review covers future research with new biologically active natural marine microbial compounds and highlights their mechanism of action with focus on chemical potential. Meticulous research in the field of bioactive compounds from marine microbes may open the gates for many prospect implications in oceanic industries as well as in the field of biomedical sciences.

The ocean is called the “mother of origin of life,” and an enormous proportion of all life on Earth exists within the oceans (Mora et al., 2011). In densely populated marine environments, space is often a limiting factor. When free space is not available, one organism grows on top of another one. This process is defined as epibiosis (Wahl et al., 2012). Adaptation to epibiosis arises via three methods: (1) tolerance; (2) avoidance; and (3) defense (Wahl et al., 2012). Overgrowth is controlled by employing either one or a combination of ecological, physical, and chemical defense mechanisms (Wahl, Jensen & Fenical, 1994), which includes the major factor, production of bioactive metabolites (Wahl & Banaigs, 1991). Studies unraveling the epibiotic chemical defense of marine microorganisms can provide insight into the development of novel ecofriendly antifouling compounds and strategies (Jensen & Fenical, 1996).

Biofouling occurrence in the marine environment is a sequential process, commencing with micro-fouling and concluding with macro-fouling. The adverse effects of biofouling and economic penalties to the marine industries are well known (Bekiari et al., 2015; Dang & Lovell, 2016). In addition, fouling is also known to cause destruction of metallic surfaces by accelerating the corrosion rate (Edyvean, Thomas & Brook, 1988). Numerous efforts are being made to control biofouling with applications of physical, chemical, biological compounds, but it is achieved to the greatest extent only with the use of anti-fouling paint coatings (Cao et al., 2011; Iorhemen, Hamza & Tay, 2016; Lade, Paul & Kweon, 2014).

**Figure 1:**
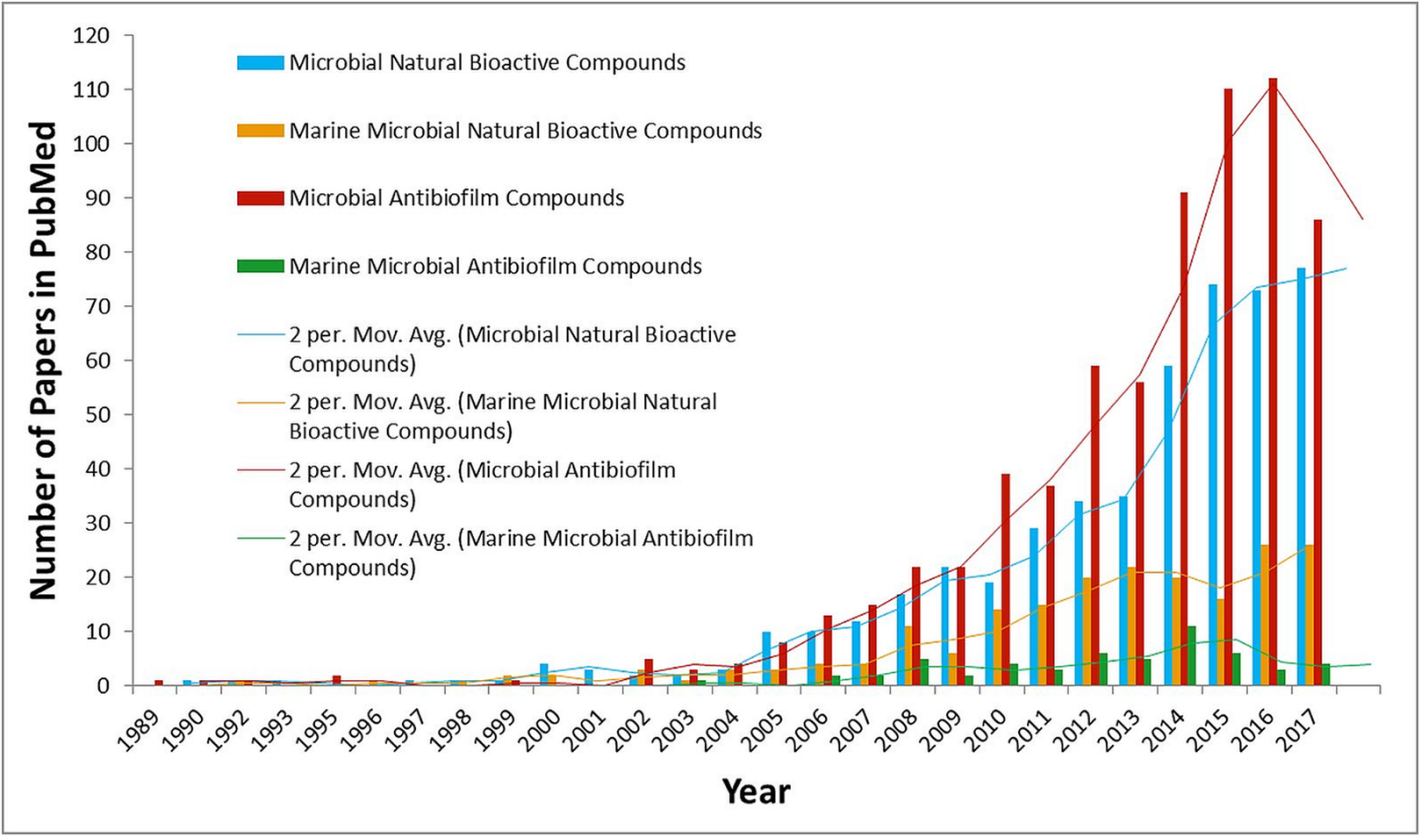
Comparative statistical data about number of publications in PubMed since 1989 till 2017. Comparative general data about the number of publications in PubMed when searching for several keywords/phrases: (1) microbial natural bioactive compounds (blue column); (2) marine microbial natural bioactive compounds (yellow column); (3) microbial anti-biofilm compounds (red column); and (4) marine microbial anti-biofilm compounds (green column). Moving average trend lines show the importance and urgent need for research concerning on marine microbial natural anti-biofouling/anti-biofilm compounds.

Use of toxic anti-foulants and its related environmental concerns have caused an increase in development of non-toxic alternatives (Callow & Callow, 2011; Nurioglu, Esteves & de With, 2015). In this perspective, studies concerning marine micro-organisms’ epibiotic chemical defense in the control of biofouling show great promise. Efforts in these areas have proven that the wide range of marine organisms such as algae, sponges, gorgonians, bryozoans, and ascidians are a potential source of anti-fouling metabolites (Ganapiriya, Maharajan & Kumarasamy, 2012; Zhang et al., 2014). However, not much detail is known to date regarding anti-biofouling/anti-biofilm natural compounds from marine microorganisms. Figure 1 demonstrates statistics about the number of publications in PubMed from 1989 until 2017 regarding this subject. These data explain the urgent need to explore the enigma of favorable marine microbes’ activities against biofouling. Since the beginning of this type of research, studies concerning marine microbial metabolites, have shown significance that is only related to therapeutic and pharmaceutical agents for various commercial and medical applications (Table 1); however, none have been identified to date, which can be viewed as the existence of potent anti-biofouling/anti-biofilm marine microbial derivatives without any synthetic modifications.

**Table 1:** List of areas commonly known for applications/uses of marine microbial natural bioactive compounds.

**Medical significance of marine microbial metabolites**	Antibacterial
	Antiviral
	Antiparasitic
	Antifungal
	Anticoagulant
	Antimutagenic
	Antihyperglycaemic
	Antitumoral
	Antiinflammatory
	Anticarcinogenic
	Antioxidant
	Taste astringent
	Photoprotection
	Immunomodulator
	Emulsifier
	Gelling agent
	Cosmetics industry
	Textile industry
	Stimulant

Enormous numbers of studies have been done to discover natural product-derived anti-foulants as a substitute for toxic anti-fouling paints (Ciriminna, Bright & Pagliaro, 2015; Omae, 2003; Qian, Xu & Fusetani, 2009; Wang et al., 2017). Epibiotic chemical defense of marine organisms have been evaluated, but some restrictions hindered those investigations. For instance, these studies were carried out in the laboratory against one or two groups of epibionts (Davis, 1991; Laudien & Wahl, 2004), and such unrealistic assay conditions do not exist in nature (Henrikson & Pawlik, 1998). This restricts the scope of studies concerning epibiotic chemical defense in marine organisms, which present a wide range of epibionts in nature. In this respect, evaluation of metabolites obtained from marine microorganisms against broad spectrum of epibionts provides better insight into epibiotic chemical defense (Penesyan, Kjelleberg & Egan, 2010).

In the real world, identification of bioactive metabolites origins is complicated as some marine invertebrates are home for a wide range of bacteria. For example, in some sponge species, bacteria contribute up to 60% of the total biomass (Mohamed et al., 2008) and in some studies they have been reported to produce bioactive metabolites similar to their host (Graça et al., 2015; Elyakov et al., 1991). Such cases are a subject of scientific debate. Several other studies have reported the isolation of bioactive metabolites from microorganisms associated with their invertebrate hosts (Kelecom, 2002; Bernan, Greenstein & Maiese, 1997; Hay, 2009). As an example, the marine sponge was the known source for the isolation of surfactant-like depsipeptides (Prasad et al., 2011; Anjum et al., 2016). Later, it was found that the bacterium *Bacillus pumilus* produces these compounds which were previously associated with just the sponge (Kalinovskaya et al., 1995).

In view of this, it is important to understand the role of associated bacteria in the production of bioactive metabolites and the importance of their association with the invertebrate host, which is still not clear at this time. However, studies to explore the relationship between anti-microbial activity and anti-fouling activities are lacking. In this context, studies to elucidate the effectiveness/isolation of various types of extracts against/from microorganisms will give a better understanding of these microorganisms’ activities.

## Survey Methodology

All peer reviewed scientific papers were used for this review article. Extensive literature searches have been performed using various literature search engines, including Science Direct and PubMed with several terms: *(1) microbial natural bioactive compounds; (2) marine microbial natural bioactive compounds; (3) microbial anti-biofilm compounds; and (4) marine microbial anti-biofilm compounds*. All relevant studies meeting search criteria were included in this review.

### Need for antibiofilm or antibiofouling bioactive compounds with green chemistry

What is green chemistry and why do we need it? Chemistry is undeniably a very obvious part of our daily lives, and new chemical developments bring new environmental complications with unexpected harmful side effects. Hence, there is continued pressure on various chemical industries to reduce chemical waste and use their creativity to develop novel synthetic methods, reaction conditions, analytical tools, catalysts, and processes under the new paradigm of “green chemistry” (Erickson, Nelson & Winters, 2012).

The term green chemistry, also known as sustainable chemistry, was introduced by Paul Anastas in 1991 (Matthews, 2000). Twelve principles of green chemistry were created that encompass a new attitude toward industrial practices and chemical syntheses (Winterton, 2016). This set of principles describes guidelines for reduction/elimination of the generation of hazardous substances while designing, manufacturing, and applying any chemical products. It is the only science that focuses on the intrinsic hazards of a chemical or chemical process. Thus, to realize more sustainable thinking and implementation of sustainability criteria in research, development, and production, educational strategies have to include green chemistry principles and indicators of sustainability (Burmeister, Rauch & Eilks, 2012; Wilson & Schwarzman, 2009).

Formation of biofilms leads to biofouling, which is a process of gradual accumulation of waterborne organisms on natural and artificial surfaces of marine environment that leads to corrosion and decline in the efficiency of moving parts (Adnan et al., 2010; Adnan, Morton & Hadi, 2011; Yebra, Kiil & Dam-Johansen, 2004). Advances in polymer chemistry in the early 1970s led to biofouling control, but it was based on the use of toxic substances such as tributyl tin (TBT) (Rouhi, 1998), copper or organic compounds (such as Sea-Nine, isothiazolone) (Yebra, Kiil & Dam-Johansen, 2004), or self-polishing copolymer anti-fouling paints, in which organotins are the biocides. So far, organotins are the most effective methods of biofouling control; however, these chemicals are toxic to aquatic environments (Yebra, Kiil & Dam-Johansen, 2004). The use of such toxic biocides presents environmental concerns involving anomalies in non-target organisms that include shell malformation in bivalves, depletion of oyster population, imposex in gastropods, and others (Park et al., 2016). TBT has been labeled as the most lethal substance to have been deliberately introduced in to the marine environment by man (Park et al., 2016). In general, the use of such toxins in marine environments poses a real threat to the marine biota (Fleming et al., 2006). Effective from January 2008, the International Maritime Organization (IMO) and Marine Environmental Protection Committee (MEPC) introduced resolutions to ban the practice of using TBT or other substances containing tin as biocides (Champ, 2003). Therefore, there is a need for the development of environmental friendly non-toxic and natural anti-biofouling agents.

### Advancement in methods used to prevent microbial biofilm and biofouling

Since removal of biofilms is a very difficult, challenging, and demanding process, a complete and cost-effective cleaning procedure should be developed (Caixeta et al., 2012) because an inappropriate cleaning strategy generally leads to biofilm formation (Garrett, Bhakoo & Zhang, 2008). A number of methods can be used to prevent microbial biofilm formation and/or biofouling. Currently, these measures fall into three economically lucrative categories: (1) physical; (2) chemical; and (3) biological methods (Cao et al., 2011). In this review, we will discuss the biological methods that are currently used. Adsorption of bioactive compounds such as bacteriocins and nisin onto food-contact surfaces reduces bacterial adhesion. Enzyme mixtures have also proven to be effective for cleaning and biofilm removal (Garrett, Bhakoo & Zhang, 2008; Stiefel et al., 2016; Guerra et al., 2005; Van Houdt & Michiels, 2010; Pimentel-Filho et al., 2014). Unique properties of Endo H (endoglycosidases) for removal of bacteria such as *Staphylococci* and *E. coli* from glass and cloth surfaces makes it useful in buffers and detergent solutions (Carpenter, Goldstein & Lad, 1995). Colanic acid-degrading enzymes, derived from a *Streptomyces* isolate, are also useful for removal and prevention of biofilm formation (Van, Bruggeman & Van, 1996).

Marine benthic organism surfaces act as potential locations for the settlement of fouling organisms, including bacteria, algae, and invertebrates (Cao et al., 2011; Pereira et al., 2002). However, at the same time these organisms present a great variety of potential defense mechanisms against fouling organisms, which include surface sloughing, possession of spines, production of mucus, low surface energy, and secondary metabolite production (Pereira et al., 2002). Secondary metabolites demonstrating anti-fouling properties opened a new perception into inhibition of overgrowth by epibionts and could possibly be used as commercial anti-biofoulants (Wahl et al., 2012). In fact, a vast range of tested anti-biofoulants have been patented. However, these anti-biofoulants have been tested under laboratory conditions, using the larvae of fouling organisms such as barnacles and bryozoans (Pereira et al., 2002; Zimmerman et al., 1997). Moreover, there are only few studies in which this test has been performed under ecological settings, in which real concentrations of metabolites found in source organisms were used (Da Gama et al., 2002). From a molecular perspective, secondary marine metabolites seem to hinder bacterial colonization and can control biofilm formation/biofouling (Kjelleberg et al., 1997). To justify and prove this statement, further investigations, including ecological and molecular tests, are required.

### Ecological role of marine bioactive compounds

Natural marine products, secondary metabolites, enzymes, lipids, and heteropolysaccharides derived from marine sources are biologically active. Moreover, they are safer, cheaper, and less toxic than existing medicines (D’Ayala, Malinconico & Laurienzo, 2008; Bergé & Barnathan, 2015). Out of 36 known living phyla, 34 are found in throughout the marine environment with more than 300,000+ known species of flora and fauna (Baskaran et al., 2017) (and still counting). However, the detailed ecological role of these marine animal-extracted secondary metabolites, which includes prevention of fouling, anti-predation, protection from ultraviolet radiation, mediation of spatial competition, and other functions (Armstrong, Boyd & Burgess, 2000) is still not clear and present areas for novel and extensive studies.

Many natural marine products are currently being tested in clinical trials, and various others are presently used for treating microbial infections (fungal, malarial, bacterial, viral, and nematode) and pain management in addition to cancer and inflammation control (Martins et al., 2014). The first marine compound to enter a human cancer clinical trial as a purified natural product was Didemnin-B from the Caribbean tunicate *Trididemnum solidum* (Ram et al., 2008). Unfortunately, secondary metabolites of macro-organisms are usually present in trace amounts, and that presents a drawback to use of the marine compounds. Due to less stock, the marine compound cannot compete with the development of widely available medicines. The developmental ways such as bioprocess engineering is currently the most important method for obtaining large quantities of beneficial secondary metabolites. This can only be accomplished by extracting and testing less investigated drug sources such as marine fungi and bacteria, which are vast untapped reservoirs of metabolic diversity (Debbab et al., 2010).

Microorganisms have some advantages such as isolation of the same compound in large-scale cultivation using biotechnological fermentations and advance bioprocessing engineering techniques with different parameters without ecological exploitation. They can also easily be genetically manipulated. There is still opportunity for an advanced scale of research and investigation to discover the potential of marine microorganisms as producers of novel drugs (Malve, 2016).

### Bioactive compounds from marine bacteria, fungi and cyanobacteria

Over the last several decades, microorganisms have been accepted as significant and unexploited resources for many unique bioactive compounds with clinical significance (Malve, 2016; Zhang et al., 2005). It is very clear that microbial diversity found in the oceans is poorly understood, and <5% of marine bacterial and fungal species are known (de Felício et al., 2015). Microbes thrive not only in the sea’s surface waters but also in the lower and immeasurable depths (Das, Lyla & Khan, 2006). For survival, soft-bodied marine organisms rely on chemical defenses from the production of bioactive secondary metabolites either by themselves or in association with microflora (Mehbub et al., 2014). These secondary metabolites may represent a diverse structure of classes that include terpenes, peptides, polypeptides, and compounds of mixed biosynthetic origin. A total of 961 new compounds from marine microorganisms were described in the year 2007 (Blunt et al., 2009). These studies and the sharp rise in numbers of these compounds indicate that the study on secondary metabolites from marine bacteria and fungi as source of new bioactive metabolites has been steadily increasing. So far, only a few anti-larval settlement compounds have been isolated and identified from bacteria (Table 2).

**Table 2:** List of few marine microbial species reported to produce bioactive metabolites with antibiofilm and antifouling activities (Abu Sayem et al., 2011; Arai, Niikawa & Kobayashi, 2013; Busetti et al., 2015; Dos Santos et al., 2010; Estrela & Abraham, 2016; Fusetani, 2011; Hong & Cho, 2013; Jiang et al., 2011; Salta et al., 2013; Satheesh, Ba-Akdah & Al-Sofyani, 2016; Scopel et al., 2014; Shao et al., 2015; Shao et al., 2011; Yang et al., 2007).

Microorganism	Bioactive compound	Biological activity	Effects against	Relevance
**Bioactive compounds isolated from marine bacteria**				
*Bacillus licheniformis*	α-D-galactopyranosyl-(1→2)-glycerol-phosphate	Antibiofilm	*Escherichia coli* and *Pseudomonas fluorescens*	Clinical
*Alteromonas* sp.	Ubiquinone-8	Antifouling	Larval settlement of barnacle *Balanus amphitrite*	Environmental
*Acinetobacter* sp.	6-bromoindole-3-carboxaldehyde	Antifouling	Larval settlement of barnacle *Balanus amphitrite*	Environmental
*Pseudomonas* sp.	Pyolipic acid	Antifouling	Larval settlement under laboratory and field experiment assays	Environmental
*Pseudomonas* sp.	Phenazine-1-carboxylic acid	Antifouling	Larval settlement under laboratory and field experiment assays	Environmental
*Pseudomonas* sp.	2-alkylquinol-4-ones	Antifouling	Larval settlement under laboratory and field experiment assays	Environmental
*Streptomyces praecox*	Diketopiperazines	Antifouling	Larval settlement under laboratory and field experiment assays	Environmental
*Streptomyces violaceoruber*	3-octa-1′,3′-dienyl-4-methylfuran-2(5*H*)-one	Antifouling	Zoospores of *Ulva pertusa*, the diatom *Navicula annexa*, and the mussel *Mytilus edulis*	Environmental
*Streptomyces violaceoruber*	3-octa-1′-enyl-4-methylfuran-2(5*H*)-one	Antifouling	Zoospores of *Ulva pertusa*, the diatom *Navicula annexa*, and the mussel *Mytilus edulis*	Environmental
*Vibrio* sp. *QY101*	Exopolysaccharide A101	Antibiofilm	*Pseudomonas aeruginosa* FRD1	Clinical
**Bioactive compounds isolated from marine fungi**				
*Letendraea helminthicola*	3-methyl-N-(2-phenylethyl) butanamide	Antifouling	Larval settlement of barnacle *Balanus amphitrite*	Industrial/Environmental
*Scopulariopsis* sp.	Dihydroquinolin-2(1*H*)-one	Antifouling	Larval settlement of barnacle *Balanus amphitrite*	Industrial/Environmental
*Letendraea helminthicola*	Cyclo (D-Pro-D-Phe)	Antifouling	Larval settlement of barnacle *Balanus amphitrite*	
*Cochliobolus lunatus*	Resorcyclic acid lactones	Antifouling	Larval settlement of barnacle *Balanus amphitrite*	Industrial/Environmental
*Emericella variecolor*	Ophiobolin K	Antibiofilm	*Mycobacterium bovis*	Clinical
*Emericella variecolor*	6-epi-ophiobolin K	Antibiofilm	*Mycobacterium smegmatis*	Clinical
*Emericella variecolor*	6-epi-ophiobolin	Antibiofilm	*Mycobacterium smegmatis*	Clinical
Unidentified marine fungus	Mevalonolactone	Antibiofilm	*Staphylococcus epidermidis*	Clinical
Marine *Penicillium* sp.	Cyclo(L-Tyr-L-Leu)	Antibiofilm	*Staphylococcus epidermidis*	Clinical/Environmental
*Penicillium commune*	Cyclo(L-Leu-L-Pro)	Antibiofilm	*Staphylococcus aureus*	Clinical/Environmental
*Cladosporium* sp.	F14 cyclo-(Phe-Pro)	Antibiofilm	*Loktanella hongkongenis*, *Micrococcus luteus* and *Ruegeria* sp.	Clinical/Environmental
*Cladosporium* sp.	cyclo-(Val-Pro) 4	Antibiofilm	*Loktanella hongkongenis*	Clinical/Environmental
*Aspergillus flavipes*	Flavipesin A 49	Antibiofilm	*Staphylococcus aureus* and *Bacillus subtilis*	Clinical
**Bioactive compounds isolated from marine algae**				
*Chondrus crispus*	(+)—Usnic acid	Antibiofilm	*Cobetia marina* and *Marinobacter hydrocarbonoclasticus*	Environmental
*Chondrus crispus*	Juglone	Antibiofilm	*Cobetia marina* and *Marinobacter hydrocarbonoclasticus*	Environmental
*Laurencia elata*	Elatol	Antifouling	*Leishmania amazonensis*	Clinical
*Halidrys siliquosa*	Organic extract	Antibiofilm	*Staphylococcus*; *Streptococcus*; *Enterococcus*; *Pseudomonas*; *Stenotrophomonas*; and *Chromobacterium*	Clinical

One of the ways of discovering novel bioactive metabolites from marine microorganisms is via isolation of new microorganisms. Research over the years has demonstrated that out of 10% of cultivable microorganisms, only 1% were found to have clinical and industrial significance (Vartoukian, Palmer & Wade, 2010). Due to the complex nature of the oceans, marine bacteria have developed sophisticated physiological and biochemical systems with which they uniquely adapt to extreme habitats and various unfavorable marine environmental conditions. They live in a biologically competitive environment with unique conditions of pH, temperature, pressure, oxygen, light, nutrients, and salinity, which is especially rich in chlorine and bromine elements. Microbes can quickly sense, adapt, and respond to their environments (Adnan et al., 2010; Adnan, Morton & Hadi, 2011) and can compete for defense and survival via the generation of unique secondary metabolites. Although the response to stress initiates the production of these compounds, they have shown value in pharmaceutical and biotechnological applications (Darvishi Harzevili & Chen, 2014).

Among all microorganisms, fungi are found to be a potentially useful source of pharmacologically active metabolites because they have the capability to adapt and survive in marine environments and to produce unique secondary metabolites. Fungi have also been widely distributed in marine habitats such as seawater, sediment, marine animals, and plants. Even though decomposition of dead plants and animal tissues has been suggested to play an important ecological role in recycling nutrients (Zhang et al., 2015), the role of marine fungi is poorly understood. Marine fungi have been isolated from different marine habitats for the investigation of natural products (Imhoff, 2016). They found to form a mutual synergetic relationship with other marine organisms such as algae, sponges, mollusks and corals, but this association and related functions are rarely known (Ding et al., 2011). Culture-based technology is used for the isolation of various fungi from marine habitats. Due to their uniqueness when compared with their terrestrial counterparts, they have also shown to be phylogenetically distant (Reich & Labes, 2017; Rédou et al., 2015). They are a promising source for new bioactive natural products with high structural diversity when compared with other microbial sources isolated from the sea. Because of their living conditions, including tolerance to salinity, high pressure, nutrition requirements, temperature variations, and competition with other microbes, they may have developed unique metabolic pathways (Samuel, Prince & Prabakaran, 2011).

The most famous and first reported group of bioactive compounds obtained from marine-derived fungi were cephalosporins (with cephalosporin C) that were isolated by Brotzu in 1945 from a marine strain of *Acremonium chrysogenum.* They had been discovered in a sewage outlet in the Mediterranean Sea close to the island of Sardinia coast, and they exhibited pronounced antibacterial activities (Silber et al., 2016; Demain & Elander, 1999). After the discovery of cephalosporin C, siccayne was isolated from *Halocyphina vilosa* and was identified as the second antibiotic from marine-derived fungi (Kupka et al., 1981). Marine-derived fungi have been widely studied for novel anti-cancer, anti-bacterial, anti-plasmodial, anti-inflammatory, and anti-viral agents (Rajasekar et al., 2012). However, very few anti-biofilm/anti-biofouling bioactive metabolites have been derived from marine fungi.

More than 270 new natural products isolated from marine fungi between 1990 and 2002 followed by 330 new metabolites between 2002 and 2006 have been reported (Imhoff, 2016). In 2009 and 2010, >472 new structures were reported. The total number of new natural products from marine-derived fungi currently exceeds 1,000, and a few of them are in preclinical and clinical trials (Gomes et al., 2015). The ecological role of these multiple compounds include prevention of fouling, competition for space/resources, protection from ultraviolet radiation, and facilitation of reproduction (Nunez-Pons & Avila, 2015). These marine-derived fungal metabolites have been proven to be promising anti-fouling/anti-biofilm agents, but still required additional investigation and implementation in oceanic-related industries.

Marine cyanobacteria (blue–green algae) are another such group of prokaryotic photosynthetic organism and are potent sources of pharmacological and industrial products having diverse structures and habitats that are known to produce various bioactive compounds (Mourelle, Gómez & Legido, 2017). The potential use of cyanobacteria has gained worldwide attention in view of their importance in agriculture, industry, pharmaceutical markets, and other areas (Singh et al., 2017). They are the richest sources of known and unique bioactive compounds, including toxins with potential for therapeutic applications (Singh et al., 2017). Marine cyanobacteria demonstrate a wide range of biological activities, including its use as biocontrol agents against various bacterial and fungal pathogens in addition to its use as a potential source of novel antibiotics. They are known to produce anti-bacterial and anti-fungal compounds (Shantanu et al., 2013), anti-cancerous and anti-neoplastic agents, and compounds that may be useful in the treatment of human immunodeficiency virus (Vijayakumar & Menakha, 2015; Swain, Padhy & Singh, 2015). Screening of cyanobacteria for potent anti-biofouling/anti-biofilm natural agents will open a new horizon for marine industries (Table 2). More than 50% of marine cyanobacteria are potentially exploitable for extracting bioactive substances, which can be effective anti-biofouling agents. Cyanobacterial blooms are rich sources of secondary metabolites with novel chemical and molecular structures (Engene et al., 2011). Exploring the efficiency of cyanobacterial products has an advantage; it can be grown in mass culture, which can be manipulated to attain optimal production of bioactive substances.

## Conclusions

Ever since the beginning of development of a civilized society, biologically active compounds, which are obtained from diverse range of microbes, have been extensively investigated. Bacterial biofilms are the dominant reason for biofouling in most commercial systems with no permanent solution toward removal of biofilms. Microbial secondary bioactive metabolites possess quite a few pharmaceutical applications. Use of ecofriendly biocides as an alternative to synthetic chemicals has recently emerged because the sole purpose of green chemistry is to either discover or to produce the best and novel chemical products that are safe for use with increased productivity. Unquestionably, our understanding of the field of microbial metabolites has significantly improved over the past several years, but there are still many steps to achieve a better understanding about the potential of marine microbial metabolites. We are in a promising era of science, which proves to be the right time; we can uncover the potential of marine microbes in the field of biofouling prevention and not in biofouling causation. In order to explore the natural anti-fouling/anti-biofilm compounds (green chemistry) and potential of marine microbes, this review will definitely draw attention to the search for bioactive metabolites that can resolve many difficulties and obstacles not only for oceanic industries, but many other industrial systems.

## Additional Information and Declarations

### Data Availability

The following information was supplied regarding data availability: This is a review article that examined peer reviewed articles available in Science Direct and Pubmed.
